# *Clostridium perfringens* sepsis after comprehensive multicourse treatment of hepatocellular carcinoma: A case report and review

**DOI:** 10.1016/j.heliyon.2024.e33279

**Published:** 2024-06-18

**Authors:** Yujuan Zhang, Yu Zhu, Yaping Han, Liyan Yang, Jingzhi Wang, Wei Cui

**Affiliations:** a Department of Clinical Laboratory, State Key Laboratory of Molecular Oncology, National Cancer Center/National Clinical Research Center for Cancer/Cancer Hospital, Chinese Academy of Medical Sciences and Peking Union Medical College, Beijing, China; b Department of Clinical Laboratory, Shanxi Bethune Hospital, Shanxi Academy of Medical Sciences, Tongji Shanxi Hospital, Third Hospital of Shanxi Medical University, Taiyuan, China

**Keywords:** Hepatocellular carcinoma, *Clostridium perfringens*, Sepsis

## Abstract

**Introduction:**

*Clostridium perfringens* sepsis is a rare but serious clinical syndrome that is typically triggered by gastrointestinal disorders. We present a case of bloodstream infection caused by *Clostridium perfringens* in a liver cancer patient after comprehensive multicourse treatment.

**Case presentations:**

The patient, a 68-year-old male, experienced nausea, decreased appetite, and abdominal distension on the 15th day after receiving comprehensive multicourse treatment and transcatheter arterial chemoembolization (TACE). During admission, he developed fever, and blood culture results confirmed the presence of *Clostridium perfringens*. The patient was discharged with improved symptoms.

**Conclusion:**

Our findings underscore the rarity of *Clostridium perfringens* sepsis. For liver cancer patients, particularly those who have undergone TACE or radiofrequency ablation and who experience post treatment fever, vigilance for *Clostridium perfringens* bloodstream infection is crucial. Timely diagnostic assessments and proactive treatment can significantly enhance the survival prospects of these patients.

## Highlights

• Here is a case of rarely bloodstream infection caused by *Clostridium perfringens* in a liver cancer patient after TACE.

## 1 Introduction

*Clostridium perfringens* (*C. perfringens*) is a gram-positive, anaerobic, rod-shaped bacterium that typically thrives in soil and the human gastrointestinal and genitourinary tracts [1]. This opportunistic pathogen can cause various conditions, including food poisoning, soft tissue infections, and distinctive gas gangrene. *C. perfringens* sepsis is a rare clinical syndrome, characterized by a low isolation rate but a high mortality rate, ranging from 27 % to 44 %. Among hospital-acquired infections, the mortality rate can soar, reaching 60.9 % [2]. The key to achieving satisfactory clinical outcomes lies in providing accurate and timely aggressive treatment. Research has demonstrated that *C. perfringens* sepsis is more likely to occur in patients with poorly controlled diabetes, liver cirrhosis, malignancies, immunocompromised conditions (such as those undergoing chemotherapy or radiation therapy, using corticosteroids), and concurrent pancreatitis [3]. The primary cause of bloodstream infections is believed to be the translocation of the gut microbiota. Currently, there are limited reports of *C. perfringens* bacteremia in patients with hepatocellular carcinoma (HCC). This article describes a case of *C. perfringens* sepsis in an HCC patient who underwent multiple comprehensive treatments. Additionally, this study offers a literature review and analysis to raise awareness among clinicians regarding the potential for *C. perfringens* sepsis during the treatment of HCC patients.

## 2 Case presentation

A 68-year-old male presented with a liver mass that had been identified during a routine physical examination on July 2022. A computed tomography (CT) scan revealed a mass located in the right posterior lobe of the liver, encompassing segments 4 and 7, with a strong likelihood of being hepatocellular carcinoma (HCC). Additionally, the left adrenal gland displayed slight thickening with an internal nodular lesion, raising suspicions of possible metastasis. The patient subsequently underwent a liver biopsy at our hospital, which confirmed poorly differentiated HCC upon pathology examination. Following this diagnosis, he underwent two rounds of percutaneous microwave ablation (MWA) for HCC. However, postprocedural assessments indicated tumor progression. Consequently, he received transcatheter arterial chemoembolization (TACE) and was started on a combination therapy regimen comprising lenvatinib hydrochloride and camrelizumab immunotherapy, completing four cycles. Subsequently, the patient started experiencing symptoms such as nausea, reduced appetite, and abdominal distension, which gradually worsened on the 15th day after he underwent digital subtraction angiography (DSA) of the mesentery, celiac trunk, and hepatic artery, in addition to left hepatic artery embolization and continuous infusion chemotherapy. As a result, he was admitted to the hospital for further evaluation and treatment.

The patient was in poor general condition and had a low mental status, diminished dietary intake, abdominal distension, and the ability to ingest only small quantities of semiliquid food. He reported intermittent episodes of diarrhea, maintained normal urine output, and occasionally experienced abdominal pain. Furthermore, he had lost 5 kg in body weight over the past two weeks. Notably, the patient had a history of alcohol consumption (at least 500 mL daily) for more than 30 years, and smoking for more than 20 years. He had been diagnosed with alcoholic cirrhosis two years prior; however, his symptoms were mild, and there were no symptoms such as liver area pain, jaundice, spider nevi, or palmar erythema before the onset of this illness.

Upon admission, the patient exhibited a body temperature of 36.1 °C, a pulse rate of 104 beats/min, a respiratory rate of 21 breaths/min, and a blood pressure of 113/81 mmHg. He maintained alertness, assumed a voluntary position, and cooperated throughout the physical examination. No signs of jaundice or hemorrhaging were apparent on the skin or mucous membranes. The superficial lymph nodes were not noticeably enlarged. The sclera exhibited a nonicteric appearance, and the conjunctiva displayed no signs of pallor. During lung auscultation, the breath sounds in both lungs were clear, with no discernible abnormal sounds. Despite abdominal distension, there was no evidence of tenderness, rebound tenderness, or muscle rigidity upon abdominal palpation. No palpable masses were detected in the abdomen, and neither the liver nor the spleen was palpable. Bilateral kidney areas did not elicit tenderness, and bowel sounds were diminished. Additionally, positive shifting dullness was observed.

Following admission, the patient received a comprehensive treatment regimen, including nutritional support, tenacissoside injection to boost immune function, thymopentin injection to enhance immunity, human albumin infusion to correct hypoalbuminemia, hypertonic saline infusion to address hyponatremia, adenosine disodium triphosphate and amino acid infusion to protect the liver and reduce bilirubin levels, and furosemide injection for diuretic therapy.

**Fig. 1:**
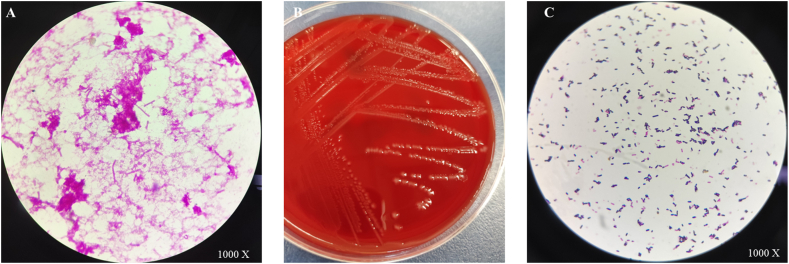
Smear of the isolated *C. perfringens*. (**A**) Peripheral venous blood culture of the anaerobic bottle, which showed the growth gram positive bacilli. (**B**) Colony morphology of the isolated *C. perfringens* on 48 hours. (**C**) Smear of the colony showed gram positive bacilli.

After 2 days in the hospital, the patient developed a fever, with a peak temperature reaching 38.5 °C. Laboratory results revealed a white blood cell count of 2.33 × 10^9^/L, neutrophil count of 1.79 × 10^9^/L, total bilirubin level of 65.2 μmol/L, direct bilirubin level of 32.3 μmol/L, indirect bilirubin level of 32.9 μmol/L, sodium ion level of 122.6 mmol/L, chloride ion level of 80.7 mmol/L, *C*-reactive protein level of 69.3 mg/L, interleukin-6 level of 1926 pg/mL, and procalcitonin level of 0.21 ng/mL. Peripheral venous blood cultures were collected, and the anaerobic bottle yielded positive results after 3 days, 22 hours, 46 minutes, with gram-positive rods observed on the smear (Fig. 1A). After 48 hours of anaerobic culture, the isolates showed a double hemolytic ring on the blood plate (Fig. 1B) and smear of the colony showed gram positive bacilli (Fig. 1C). The isolate was identified as *C. perfringens* using the Vitek 2 compact system.

The patient underwent intravenous therapy with cefoperazone and sulbactam for 5 days, resulting in the normalization of body temperature and slight improvement in symptoms. However, due to significant abdominal distension attributed to ascites, the patient underwent abdominal paracentesis for drainage, which resulted in the removal of pale yellow-colored ascitic fluid. Subsequently, the patient experienced relief from abdominal distension, gradual improvement in clinical signs, absence of fever, improved appetite, and normal bowel movements, and was subsequently discharged from the hospital.

Patients with primary hepatocellular carcinoma (HCC) often have compromised immune systems and impaired liver function, which increases their vulnerability to infections, including those caused by *C. perfringens*. A comprehensive search conducted until September 2023 revealed a total of 13 studies, comprising 7 published in the English language, 3 published in Japanese, and 3 published in Chinese, that reported cases of *C. perfringens* bloodstream infections in patients with primary HCC. These studies were identified through searches of the PubMed and Wanfang databases, using the keywords “HCC” and “*Clostridium perfringens*”. 7 English case reports were included to analyze for their sufficient information [[4], [5], [6], [7], [8], [9], [10]]. The study summarized various clinical characteristics, including age, sex, treatment modalities, antibiotics administered, and patient outcomes, which are presented in Table 1. Notably, 6 out of 8 patients (75 %) were aged over 65 years, indicating a higher prevalence among older individuals. Among the patients, 4 underwent TACE, 3 after ablation therapy, and 1 following partial hepatectomy. Additionally, 3 out of 8 patients (37.5 %) died, primarily due to intravascular hemolysis (Table 1).

**Table 1:** Cases of *C. perfringens* sepsis and HCC published in English until Sep. 2023.

Author	Nationality	Time	Age/Sex	Primary affection	Underlying disease	Treatment	Post-treatment time (PTT)	Initial symptom	Antibiotic use	Positive time	Blood culture	Liver abscess	Outcome
Lauren B. Gerson et al.	San Francisco, CA	1994	83/male	HCC	no	chemoembolization	one day	fever 38.6	Cefotetan	2d	Clostridium perfringens	NA	died after 4h
Jing-Huan Li et al.	China	2015	71/male	HCC	no	TACE	two days	high fever (39.4 °C) accompanied with chill	piperacillin/tazobactam combined with Levofloxacin	2d	Clostridium perfringens	YES	survival
Takaaki Yoshikawa	Japan	2018	70/male	HCC	myocardial infraction	one week after TACE, one day after radiofrequency ablation (RFA)	one week/one day	dyspnea and a high fever (41.2 °C) with shivering	penicillin G and synergic clindamycin	NA	Clostridium perfringens	YES	survival
Haruki Uojima et al.	Japan	2019	83/male	HCC	reflux esophagitis, hypertension, and pancreatic carcinoma and he underwent pylorus-preserving pancreaticoduodenect-omy approximately 5 years ago.	TACE	six days	Fever (39.0 °C) and hematuria with jaundice	cefmetazole 3 g daily to prevent infection; anti-biotic therapy (piperacillin/tazobactam 4.5 g and clin-damycin 600 mg) combined with surgical debridement	NA	Clostridium perfringens	YES	died after 6h
Ryoga Hamura	Japan	2020	69/male	HCC	chronic hepatitis B	anterior segmentectomy of the liver and cholecystectomy	15 days	fever	CT guided percutaneous abscess drainage; Ciprofloxacin and Clindamycin	NA	Clostridium perfringens	YES	survival
Ming-Hung Wang	China, Taiwan	2021	63/female	HCC after 2 sessions of TACE	diabetes mellitus, hypertension, chronic hepatitis B-related cirrhosis	RFA	6 hours	fever (39.6 °C) chills, dyspnea and abdominal pain	aggressive fluid resuscitation, intravenous Flomoxef, Ultra-sound-guided aspiration to the lesions, coagulopathy correction.	3d	Clostridium perfringens	YES	survival
Jiang Guo et al.	China	2022	62/male	HCC	HBV-related cirrhosis and left lateral segmentectomy 3 years prior	one week after TACE, one day after microwave ablation (MWA)	one week/one day	fever(41.2 °C) and abdominal pain	intravenous cefuroxime; 4 hours later switched to combination of meropenem and vancomycin	16h	Clostridium perfringens	YES	16h later died
case	China	2023	68/male	HCC	no	TACE	15 days	fever (38.5 °C) and abdominal pain	intravenous cefoperazone sodium and sulbactam sodium	3 days	Clostridium perfringens	NA	survival

## 3 Discussion

*C. perfringens* is a common intestinal bacterium that can cause infections under certain conditions. These circumstances include biliary obstruction and diabetes, which compromise the immune response and the self-repair capacity of the liver. Consequently, individuals with these conditions become more susceptible to *C. perfringens* infections. Notably, cancer and immunosuppression have consistently been identified as risk factors for *C. perfringens* infections, particularly in patients with hepatocellular carcinoma (HCC) and cholangiocarcinoma [2]. Malignant tumors have the potential to disrupt mucosal barriers, thereby increasing the likelihood of bloodstream infections [11]. Furthermore, patients with liver cancer often undergo various treatments such as surgery, radiation therapy, chemotherapy, or embolization, all of which can weaken the immune system and potentially cause damage to the gastrointestinal mucosa. This compromised integrity of the gastrointestinal mucosa can facilitate entry of intestinal bacteria into the bloodstream, ultimately leading to *C. perfringens* bloodstream infections.

In this case report, we documented the occurrence of a *C. perfringens* bloodstream infection in a patient with HCC following multiple rounds of comprehensive treatment. Although the incidence of *C. perfringens* bloodstream infections in liver cancer patients is exceedingly rare, Chien-Chang Yang et al. conducted a comprehensive review of ten years of blood culture data from a large hospital and identified an annual incidence rate of less than one in a million for *C. perfringens* [2]. Despite its extreme rarity, *C. perfringens* bacteremia is associated with a notably high mortality rate, ranging from 27 % to 44 %. In our analysis of eight patients, we observed that mortality occurred in patients who experienced severe intravascular hemolysis and postoperative hypotensive shock, constituting 3 out of 8 patients (37.5 %).

All eight HCC patients presented with fever after developing *C. perfringens* bloodstream infection. Half of these patients had previously undergone TACE, a procedure that can lead to liquefaction necrosis of tumor tissue. This necrotic tissue creates a relatively acidic and hypoxic environment that promotes the growth of anaerobic bacteria. Bacterial infections can occur through either the biliary or gastrointestinal tract, with bacteria entering the necrotic tumor and potentially causing liver abscesses or bloodstream infections. Liver abscesses are known complications following TACE, and our study revealed that 6 out of 8 patients with *C. perfringens* bloodstream infections also had concurrent liver abscesses. Several factors, including a history of previous abdominal surgery, multiple treatment regimens, advanced age, and chemotherapy-induced immunosuppression, can collectively contribute to an immunocompromised state that is more susceptible to infections. In this context, *C. perfringens* can proliferate in occluded and necrotic areas created by TACE. Furthermore, disruption of the gastric and intestinal barriers, often secondary to HCC, is one of the most common risk factors for both *C. perfringens* bacteremia and hemolysis [5].

*C. perfringens* is classified into seven types, A, B, C, D, E, F and G, according to its ability to produce α--toxin, enterotoxin, and necrotizing enterotoxin [1]. *C. perfringens* can also produce phospholipase C, which can break down red blood cell membranes, resulting in hemolysis. This hemolysis leads to elevated levels of bilirubin, potassium, lactate dehydrogenase (LDH), and other biochemical markers. Intravascular hemolysis is a severe complication that occurs in approximately 7–15 % of patients and it has a high mortality rate ranging from 70 % to 100 % [12]. Once *C. perfringens* bloodstream infection is suspected, aggressive measures, including prompt surgical drainage of abscesses, timely administration of appropriate intravenous antibiotics, multidisciplinary circulatory support, and early laboratory investigations for intravascular hemolysis, are crucial. Suitable antibiotics for treating *C. perfringens* infection include penicillin G, metronidazole, and piperacillin/tazobactam, as some strains have shown resistance to clindamycin [11].

Although case reports are valuable for providing individualized clinical information, they also have some limitations. This case report is based on a retrospective data analysis and lacks a control group, which makes it difficult to determine whether different treatments are associated with *C. perfringens* bloodstream infection, and we are unable to exclude the influence of other unknown or unconsidered factors on the research results.

## 4 Conclusion

For liver cancer patients, especially those undergoing procedures such as TACE or radiofrequency ablation, it is imperative to maintain a high level of suspicion for *C. perfringens* bloodstream infection when evaluating patients with post treatment fever. Alongside considering postembolization syndrome, performing early and comprehensive examinations, pursuing aggressive treatment approaches, and promptly administering appropriate antibiotics can markedly improve patient survival rates. Timely intervention is crucial in the management of this potentially life-threatening condition.

## Ethics statement

Written informed consent was obtained from the patient for the publication of all images, clinical data and other data.

## Funding

This study was supported by 10.13039/501100003345CAMS Innovation Fund for Medical Sciences (CIFMS) (Grant No. 2021-I2M-C&T-B-056). The funders were not involved in the study design, collection, analysis, interpretation of data, writing of this article, or the decision to submit it for publication.

## Data availability statement

Data associated with this case report isn't been deposited into a publicly available repository. Data included in article/supplementary material/referenced in article.

## CRediT authorship contribution statement

**Yujuan Zhang:** Writing – review & editing, Writing – original draft. **Yu Zhu:** Formal analysis. **Yaping Han:** Data curation. **Liyan Yang:** Data curation. **Jingzhi Wang:** Resources, Investigation. **Wei Cui:** Supervision.

## Declaration of competing interest

The authors declare that they have no known competing financial interests or personal relationships that could have appeared to influence the work reported in this paper.
